# Early peritoneal-scrotal leakage in a patient submitted to peritoneal dialysis demonstrated by dynamic peritoneal 99mTc-phytate scintigraphy

**DOI:** 10.1590/S1677-5538.IBJU.2014.0639

**Published:** 2015

**Authors:** Andrés Martínez-Esteve, Francisco Javier García-Gómez, Juan Ignacio Cuenca-Cuenca, Juan Luis Tirado-Hospital

**Affiliations:** 1 Departamento de Medicina Nuclear, Hospital Virgen del Rocío Universitario, Sevilla, España

A 77 year old male with history of renal lithiasis leading to right nefrectomy and end stage renal disease (ESRD) secondary to possible vascular nephropathy and focal segmental glomerulosclerosis diagnosed in 2009, started ambulatory peritoneal dialysis (APD) due to clinical decline. Insufficient drainage of the peritoneal dialysis solution with progressive bilateral testicular edema (more severe in right side) was observed from the first APD session. As a consequence, the patient needed temporarily hemodialysis. In order to diagnose a patent peritoneal-vaginal duct, a dynamic 99mTc-Phytate scintigraphy was performed after introduction of peritoneal dialysis solution (600, 1200 and 2000 mL) labelled with 74 MBq of 99mTc-Phytate in the abdominal cavity. A total of 60 images (30 seconds per image) were acquired and a hypogastric uptake of radiotracer was observed (Figure 1 - Panel a). Right inguinal and scrotal uptake was observed only after performing the Valsalva's maneuver (Figure 1 - Panel b). Delayed sectorial images (Figures 2 and 3) were acquired following the peritoneal drainage period (250 seconds per image), revealing the presence of peritoneal dialysis solution in both inguinal canal and right scrotal area (Arrow and head arrow). Due to the peritoneal-vaginal leakage an inguinal hernioplasty with raquideal anesthesia was performed.

**Figure 1:**
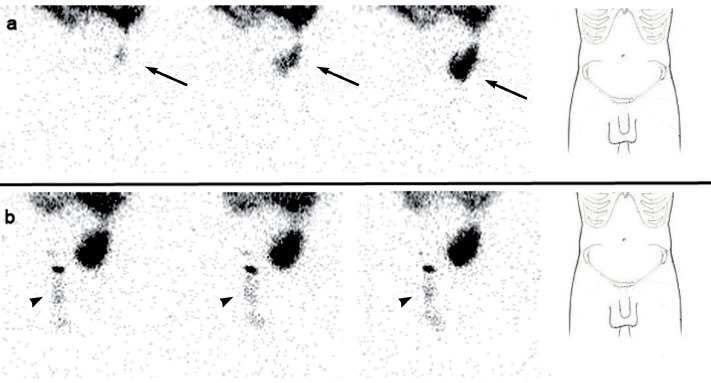
Dynamic peritoneal 99mTc-Phytate scintigraphy. Panel a: Images acquired during the filling of peritoneal dialysis solution (600, 1200 and 2000 ml) labeled with 74 MBq of 99mTc phytate, revealing a hypogastric uptake of radiotracer (arrow), not reaching the inguinal canal or the scrotal area. On the right side, anatomical reference contour. Panel b: Inguinal and right scrotal uptake of radiotracer (head arrow) after Valsalva's maneuver. On the right side, anatomical reference contour.

**Figure 2:**
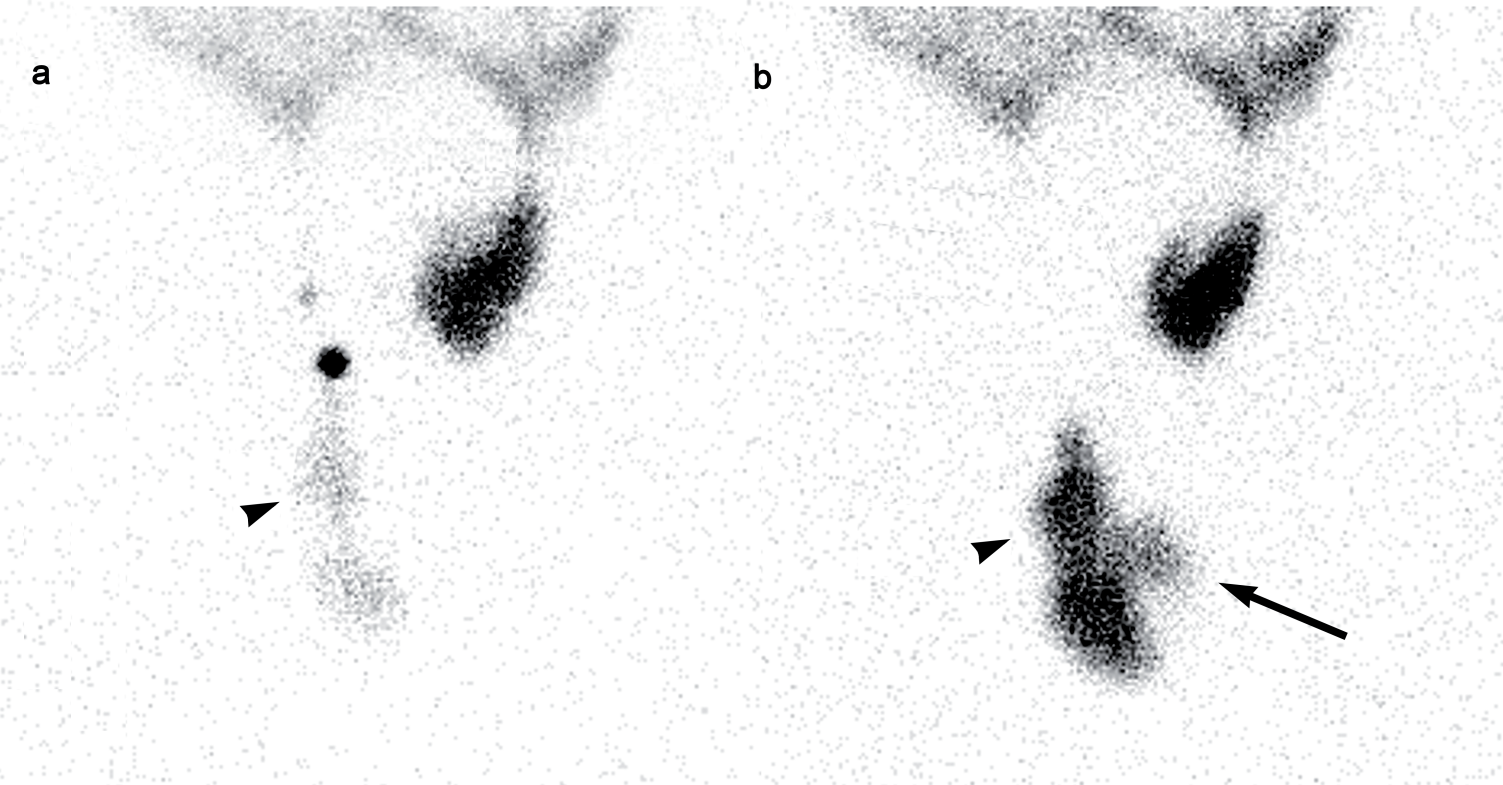
Sectorial peritoneal 99mTc-Phytate scintigraphy. Panel a: post-filling period image revealing the right inguinal and scrotal uptake of radiotracer (head arrow). Panel b: Post peritoneal lavage image, showing the appearance of left inguinal canal uptake of radiotracer (arrow).

**Figure 3:**
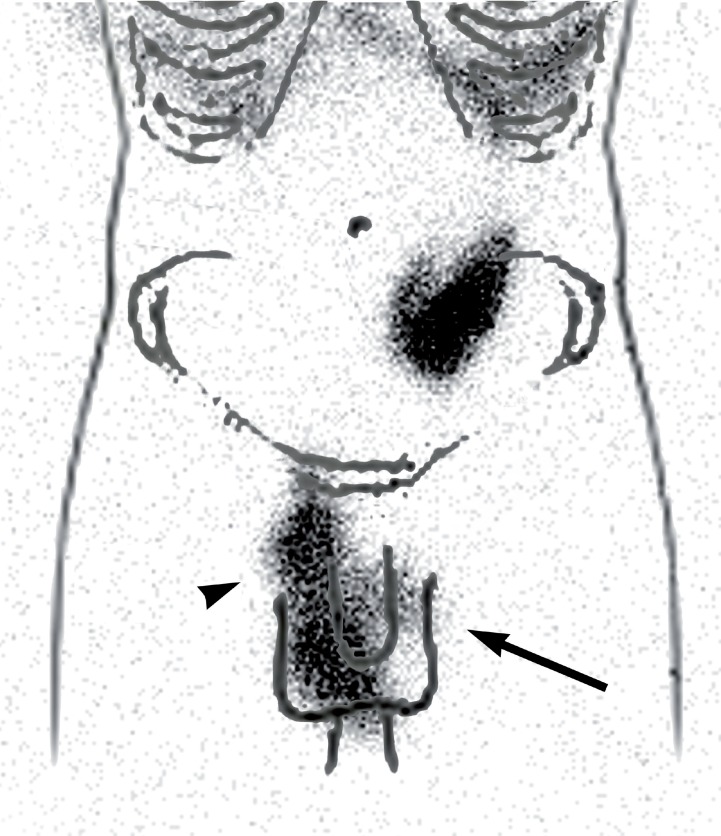
Sectorial peritoneal 99mTc-Phytate scintigraphy post-peritoneal lavage image over anatomical reference contour showing the right inguinal and scrotal uptake of radiotracer (head arrow) and left inguinal uptake (arrow).

The peritoneal-scrotal dialysate leakage is the major non-infectious catheter-related complication in patients receiving APD, caused by increased intraperitoneal pressure and the loss of integrity of the peritoneal membrane, although the most common cause is fluid extra-vasation from an indirect hernial sac or patent peritoneal-vaginal duct (1). Pleural, abdominal or genital dialysate leaks tend to develop during the first year of APD, while early leaks after catheter insertion are usually observed in the first 30 days of APD (2).

Several diagnostic methods including intraperitoneal infusion of contrast, abdominal x-ray, computed tomography or peritoneal scintigraphy are employed when the leak diagnosis is uncertain. The peritoneal scintigraphy has proven to be a useful tool for the diagnosis of the peritoneal leakage (2,3), being an effective method for identifying structural abnormalities and localization of the source of the leak.

## References

[1] Engeset J, Youngson GG (1984). Ambulatory peritoneal dialysis and hernial complications. Surg Clin North Am.

[2] Leblanc M, Ouimet D, Pichette V (2001). Dialysate leaks in peritoneal dialysis. Semin Dial.

[3] Tokmak H, Mudun A, Türkmen C, Sanli Y, Cantez S, Bozfakioğlu S (2006). The role of peritoneal scintigraphy in the detection of continuous ambulatory peritoneal dialysis complications. Ren Fail.

